# High Level of Blood Eosinophils and Localization of Bronchiectasis in Patients with Severe Asthma: A Pilot Study

**DOI:** 10.3390/jcm12010380

**Published:** 2023-01-03

**Authors:** Vitaliano Nicola Quaranta, Silvano Dragonieri, Maria Rosaria Vulpi, Nunzio Crimi, Claudia Crimi, Pierachille Santus, Francesco Menzella, Corrado Pelaia, Giulia Scioscia, Cristiano Caruso, Elena Bargagli, Nicola Scichilone, Giovanna Elisiana Carpagnano

**Affiliations:** 1 Department of Basic Medical Sciences, Neuroscience and Sense Organs, Section of Respiratory Disease, University “Aldo Moro” of Bari, 70124 Bari, Italy; 2 Department of Internal Medicine and Specialistic Medicine, Section of Respiratory Diseases, University of Catania, 95100 Catania, Italy; 3 Department of Clinical and Biomedical Sciences, Division of Respiratory Diseases, “L.Sacco” University Hospital, Università degli Studi di Milano, 20100 Milan, Italy; 4 Pulmonology Unit, S. Valentino Hospital, Montebelluna (TV), AULSS 2 Marca Trevigiana, 31044 Montebelluna, Italy; 5 Department of Health Sciences, Section of Respiratory Disease, University “Magna Græcia” of Catanzaro, 88100 Catanzaro, Italy; 6 Department of Medical and Surgical Sciences, University Hospital Policlinico Riuniti of Foggia, University of Foggia, 71100 Foggia, Italy; 7 Department of Medical and Surgical Sciences, UOSD DH Gastroenterology, Polyclinic Foundation a. Gemelli IRCCS Cattolica University del Sacro Cuore, 00186 Rome, Italy; 8 Respiratory Diseases and Lung Transplant Unit, Department of Medical and Surgical Sciences and Neurosciences, University of Siena, 53100 Siena, Italy; 9 PROMISE Department, University of Palermo, 90121 Palermo, Italy

**Keywords:** asthma, bronchiectasis, eosinophilia

## Abstract

Background. Severe asthma and bronchiectasis are heterogeneous diseases that frequently coexist. The location of bronchiectasis is generally determined by specific underlying pathophysiological mechanisms. The aim of this study was to determine whether in a population suffering from both severe asthma and bronchiectasis there was a correlation between eosinophilic inflammation and localization of bronchiectasis. Methods. We enrolled 41 patients with coexisting bronchiectasis from eight different severe asthma center outpatient clinics and collected the following data: baseline characteristics, Asthma Control Test, Asthma Control Questionnaire, IgE level, blood count, high-resolution computed tomography and bronchiectasis-related parameters, skin prick test, FeNO50 and flow-volume spirometry. The study was retrospectively registered. Results. The presence of eosinophils > 1000 cells/μL was related to distribution of lower pulmonary bronchiectasis (9.1% upper lobes vs. 53.3% lower lobes, *p* = 0.014). Indeed, the presence of eosinophilic counts > 1000 increased the probability of lower localization of bronchiectasis compared to upper lobes (ODD 0.088 (0.010–0.772), *p* = 0.028). Conclusions. An increase in blood eosinophils > 1000 cells/μL seems to be associated with lower preferential localization of bronchiectasis with sparing of the upper lung lobes. This could represent a new potential radiological phenotype that could have a dedicated therapeutic strategy in the future.

## 1. Introduction

Severe asthma and bronchiectasis coexist in a far from rare way. Among patients with bronchiectasis, asthma was reported in 3–8% of patients [1], whereas bronchiectasis appears to be present in over 24% of patients with severe asthma [2]. However, the above relationship seems to go beyond mere epidemiological association. It is well-known that asthma and bronchiectasis are very heterogeneous diseases with different phenotypes and with likely different underlying pathophysiological causes.

Among asthmatic patients there are specific populations that require a specific approach, depending on the presence of specific inflammatory phenotypes [3,4]. An inflammatory eosinophilic phenotype is more common in asthma [5], but a neutrophil inflammatory phenotype is also present predominantly in a subset of patients with severe asthma, especially in obese patients with a history of smoking, and with exposure to irritants or frequent viral infections [6]. Moreover, the complexity of bronchiectasis is equally well-known. In this regard, it is possible to identify an inflammatory eosinophilic endotype [7] in addition to the more common neutrophilic endotype [8]. Recent advances in the endotyping, genetics and disease heterogeneity of bronchiectasis have contributed to affirm its complexity [9]. Interestingly, some forms of bronchiectasis are mainly localized in the upper lung lobes (cystic fibrosis-CF, sarcoidosis), others in the middle lobe or lingula (atypical mycobacterial infection) and even in the lower lobes (chronic aspiration, primary ciliary dyskinesia, a1-antitrypsin deficiency-allergic bronchopulmonary aspergillosis [ASBA]). The location of bronchiectasis is generally determined by specific underlying pathophysiological mechanisms. Therefore, the correct identification of a radiological phenotype can help to categorize patients who deserve targeted treatments [10]. To date, there are no approved therapies for the condition other than for bronchiectasis caused by cystic fibrosis [9].

Similarly, there are no current specific therapeutic indications for patients with severe eosinophilic asthma and the coexisting presence of bronchiectasis [11]. The identification of a potential subset of patients with severe eosinophilic asthma who have a preferential radiological localization of bronchiectasis can help to improve the understanding, and potentially the treatment, of these two complex diseases.

Based on the above, the aim of the current study was to assess whether, in a population suffering from severe uncontrolled asthma and coexisting bronchiectasis, the presence of high values of blood eosinophilia are associated with a preferential location of non-CF and non-ASBA bronchiectasis in the lung.

## 2. Materials and Methods

### 2.1. Patients

A total number of 41 patients participated in the present study. Subjects were enrolled from among those attending eight Severe Asthma Center outpatient clinics (SA centers) in the following eight Italian cities: Siena, Bari, Foggia, Catania, Palermo, Milano, Rome and Reggio Emilia. 

The inclusion criteria of the study were: confirmed diagnosis of severe uncontrolled asthma with eosinophilic phenotype, being under therapy according to GINA steps 4–5 [3], not being under current and previous biological therapy and having coexisting presence of non-CF and non-ASBA bronchiectasis. 

Eosinophilic phenotype was defined by the presence of peripheral eosinophilic counts > 150 cells/μL and severe asthma was defined as “asthma requiring treatment with high-dose inhaled corticosteroids (ICS) plus a second controller (and/or systemic corticosteroids) to avoid that it becomes uncontrolled or that it remains uncontrolled despite this therapy” [12]. 

The morphological criteria for bronchiectasis on CT scans include bronchial dilatation with respect to the accompanying pulmonary artery, lack of tapering of bronchi, and identification of bronchi within 1 cm of the pleural surface. Clinically significant bronchiectasis was defined as the presence of both bronchial dilatation on computed tomography (CT) and the presence of clinical syndrome characterized by cough, sputum production and/or recurrent respiratory infections. [11]. The subregional bronchi were measured. Two independent radiologists confirmed the presence of bronchiectasis on CT and their localization in the lung.

We considered distribution of bronchiectasis in the lung as follows: upper lobes, intermediate (medium lobe and lingula) and lower lobes.

Patients with other pulmonary comorbidities such as CF, bronchiectasis due to CF, allergic bronchopulmonary aspergillosis autoimmune disease (ABPA) with pulmonary involvement, positive skin prick test for Aspergillus Fumigatus, pulmonary mycobacteriosis, former pulmonary tuberculosis, pulmonary fibrosis and any type of immunoglobulin deficiency were excluded from the study.

### 2.2. Study Design

We conducted a transversal retrospective multicenter study. Patients were enrolled from 1 January 2020 to 31 December 2020. Data were collected from all patients during a one day visit.

This study was carried out according to the principles of the Declaration of Helsinki, was approved by the local Ethics Committee of the “Riuniti” Hospital of Foggia (Institutional Review Board approval number 17/CE/2014), and all recruited patients gave their written informed consent.

The following data were obtained: baseline characteristics, detailed clinical history with all asthma-related parameters (Asthma Control Test [ACT], Asthma Control Questionnaire [ACQ], IgE level, complete blood count) and bronchiectasis-related parameters (number and location). Subsequently, skin prick test, FeNO50, flow-volume spirometry and high-resolution computed tomography (HRCT) were performed.

### 2.3. Skin Prick Test

The skin prick test (SPT) was performed for a panel of inhalant allergens as previously described for common aero-allergens (Lofarma, Milan, Italy) and was considered positive when eliciting a wheal diameter ≥ 3 mm, using negative (saline) and positive (histamine 10 mg/mL) controls for interpretation [13].

### 2.4. Lung Function

All lung function measurements were made according to European Respiratory Society/American Thoracic Society standardization [14]. Post-bronchodilator Forced Expiratory Volume in 1s (FEV1) and Forced Vital Capacity (FVC) were measured using a spirometer (Masterlab Jaeger, Hoechberg, Germany) and the best value of three maneuvers was expressed as a percentage of the predicted normal value [15].

### 2.5. Measurement of FeNO50

FeNO measurement was performed according to guidelines [16]. The measurement range is 0–600 ppb. FeNO50 was measured using a restricted breathing technique that employed expiratory resistance and positive mouth pressure to close the veil and exclude nasal NO and a constant expiratory flow of 50 mL/s. Repeated exhalations were performed until three plateaus agreed within 5% of the difference between observations [17].

### 2.6. Statistical Analysis

The Kolmogorov–Smirnov test was used to evaluate the normal distribution of data. Continuous parameters with normal distribution are reported as mean ± standard deviation, while those without normal distribution are reported as median (interquartile range). Categorical values were analyzed using the chi-square test or Fisher’s exact test as appropriate and were reported as n (%). Continuous variables were compared by Student’s t-test for independent samples for normally distributed data or by Mann–Whitney U test for non-normally distributed data.

Univariate binomial logistic regression analyses were performed to define the probability to have upper lung localization of bronchiectasis.

Significance values were assumed for *p* < 0.050. All statistical analyses were performed using SPSS for Windows 23.0 (SPSS, Chicago, IL, USA).

## 3. Results

### 3.1. Baseline Characteristics of the Population

We enrolled 41 patients with average age of 63.36 ± 11.62 and median of 67.00 (56.75–71.00). A total of 46.3% were females and only 7.3% were current smokers. The median BMI was 25.30 (21.02–29.27).

### 3.2. Asthma Parameters

The median duration of asthmatic disease was 39.00 (20.00–54.00) years. All patients were in therapy according to Step 4 or 5 GINA and presented an uncontrolled asthma with median ACQ of 3.20 (2.92–3.78) and median ACT of 16.00 (11.25–18.00). The median eosinophilic values were 625.00 cells/μL (365.00–1140.00). A total of 70.7% of patients had eosinophilia values > 500 cells/μL, whereas 11 out of 41 patients (26.8%) had eosinophilic count > 1000 cells/μL. Concerning lung function, median FEV1% pred was 63.00 (36.17–75.75) and median FVC% pred was 82.15 (76.75–92.00). Moreover, 58.5% (n = 24) of patients had nasal polyps and 53.7% (n = 22) had chronic rhinosinusitis.

### 3.3. Bronchiectasis Parameters

In 82.9% of cases, the patients presented bilateral bronchiectasis; in 78% of the cases they presented a basal location, in 56% of cases a localization at the average level or lingula level, and 41.5% of the cases were located at the level of higher pulmonary segments (Table 1).

### 3.4. Comparison between Two Groups: Eos > 1000 cells/μL vs. Eos ≤ 1000 cells/μL

The population was divided into two subgroups based on the 75th percentile of eosinophilic count: those with values less than or equal to the 75th percentile (n = 30) and those with values greater than the 75th percentile (n = 11). Therefore, we observed that the presence of an eosinophilia cutoff of 1000 cells/μL described a different distribution of pulmonary bronchiectasis. The population was therefore divided into two subgroups: the first composed of patients with eosinophilia > 1000 cells/μL and the second of patients with eosinophilia ≤ 1000 cells/μL.

Patients with eosinophilic counts ≤ 1000 cells/μL (n = 30) had a median age of 65.5 years (59.0–73.0), which was similar to that of the 11 patients with eosinophil counts > 1000 cells/μL, who had a median age of 54.0 years (45.0–74.0). Sex, BMI and asthma-related comorbidities were comparable. Similarly, the median ACT values were similar between patients with eosinophils > 1000 and those with a value ≤ 1000 (14.5 vs. 17), as was also the case with ACQ (3.15 vs. 3.0), asthma disease duration (47.99 vs. 26), %FEV1 (57.30 vs. 52), %FVC (80 vs. 80.30), IgE (253 vs. 252), FeNO50 (76 vs. 38) and WBC (76,205.50 vs. 7300.00). Furthermore, we verified that there were no significant differences between individuals with eosinophils > 1000 and those with eosinophils ≤ 1000 in terms of nasal polyposis and chronic rhinosinusitis.

Regarding bronchiectasis localization, it emerged that the presence of eosinophilia > 1000 cells/μL was related to distribution of lower pulmonary bronchiectasis (9.1% upper lobes vs. 53.3% lower lobes, *p* = 0.014) (Table 2).

In the group of patients with eosinophilia > 1000 (n = 11), only 9.1% (n = 1) had superior localization of bronchiectasis, while 90.9% (n = 10) had inferior localization. The group of patients with eosinophilia ≤ 1000 (n = 30) included 53.3% (n = 16) presenting an upper localization of bronchiectasis and 46.7% (n = 14) presenting a lower localization. The difference in the distribution of bronchiectasis between the two groups was statistically significant (*p* = 0.014)

Among the patients enrolled in the study, 24.4% (n = 10) had widespread localization of bronchiectasis while 12.2% (n = 5) patients presented localization of bronchiectasis only in the lower lung lobes. Within these two subgroups of patients (n = 15), 33.3% (n = 3) had eosinophil counts > 1000. High eosinophil count > 1000 was present in 2 (18.2%) patients with widespread bronchiectasis and in 1 (25.0%) patient with only baseline bronchiectasis). The distribution between the two subgroups was not statistically significant (*p* = 0.242).

### 3.5. Odds of Upper Lung Localization of Bronchiectasis

The presence of eosinophilic counts > 1000 cells/μL was a significant predictor that increased the probability of lower localization of bronchiectasis with a saving of the upper lobes (ODD 0.088 (0.010–0.772); *p* = 0.028). The variable “Upper Localization Bronchiectasis” included all patients who had bronchiectasis in the upper lobe, regardless of any other site. The other patients instead did not present bronchiectasis in the upper lobe, but presented inferior and/or medio/lingular bronchiectasis (see Table 3).

Univariate binomial logistic regression showed that there were no other parameters and variables (i.e., asthma-related comorbidities, duration of asthma disease, functional parameters, ACT, ACQ, FeNO50) related to bronchiectasis localization. Moreover, we verified that nasal polyposis (*p* = 0.501) and chronic rhinosinusitis (*p* = 0.181) were unable to predict upper or lower localization of bronchiectasis (see Table 3).

## 4. Discussion

To the best of our knowledge, our study showed for the first time a potential new radiological phenotype within a population of 41 patients with severe eosinophilic asthma and coexisting non-CF and non-ASBA bronchiectasis.

In fact, in our population, the presence of eosinophilia > 1000 cells/μL was related to distribution of lower pulmonary bronchiectasis (9.1% upper lobes vs. 53.3% lower lobes *p* = 0.014). The presence of eosinophilic counts > 1000 cells/μL reduced the probability of upper lobes localization of bronchiectasis (ODD 0.088 [0.010–0.772], *p* = 0.028).

Analysis of the literature reveals that few authors have previously investigated eosinophilic inflammation in bronchiectasis, which is traditionally considered a typical neutrophilic inflammatory pattern [18]. Therefore, the relationship between eosinophil pathway and severity of disease in terms of exacerbations remains controversial [19,20]. Very recently, Oriano et al. [21] hypothesized the presence of T2-high endotype in asthma patients with bronchiectasis. Interestingly, patients with Th2 inflammation showed a more severe disease, in terms of dyspnea, respiratory function and quality of life. Therefore, Th2-high endotype as a treatable trait was proposed, with relevance to a targeted therapy for bronchiectasis patients [21].

Furthermore, some radiological phenotypes of bronchiectasis with preferential basal localization have been previously described [10]. In some cases, the preferential cause of the lower lung site is due to airways shape. For instance, in patients with chronic aspiration of gastric material, aspiration of gastric contents is localized for gravitational reasons mainly to the lower lung lobes and can cause inflammation of the airways and weakening of the wall, which can result in bronchiectasis [22]. Moreover, in primary ciliary dyskinesia (PKD), the abnormality of the dynein arms of the epithelial cilia leads to an inability to perform adequate ciliary movement, thus compromising muco-ciliary clearance. In this way, the mucus accumulates mainly in the lower airways for gravitational reasons and patients are predisposed to repeated infections, airway damaging and bronchiectasis [23].

In other cases, the reason for the basal localization of bronchiectasis is to be found primarily at the vascular level. Actually, in some patients with severe asthma, the imbalance between proteases and antiproteases can lead to the development of bronchiectasis and the action of neutrophil elastase has been analyzed as one of the main causes of inflammation. Neutrophilic elastase (NE) is one of the major proteolytic enzymes involved in inflammatory response and tissue damage. Airway infection, particularly PA, is usually considered to trigger this dysregulated inflammatory pattern, which persists even after successful antibiotic treatment. Alpha-1-antitrypsin is a neutrophilic elastase inhibitor (an antiprotease). In the lungs, alpha-1-antitrypsin deficiency increases the activity of neutrophilic elastase, which facilitates tissue damage, causing not only emphysema, but also bronchiectasis [18]. The damage is mainly localized at the level of the lung bases as they are more perfused and therefore exposed to the excessive activity of neutrophil elastase [24].

In our group of patients with eosinophils > 1000 cells/μL, the distribution of bronchiectasis appears to have an apical-basal gradient. Only in 9% was bronchiectasis localized at the level of the upper lobes, while in 54.5% it was at the level of the middle lobe/lingula and in 72.7% at the level of the lung bases. We may suggest a pathogenetic model like that of alpha-1 antitrypsin deficiency, in which an excess of inflammatory mediators exerts its action mainly at the level of the most perfused areas of the lung, thus safeguarding the less perfused upper lung lobes. Therefore, chronic inflammation weakens the bronchial wall and obstruction leads to bronchial dilation.

Additionally, in our patients with marked blood eosinophils, we may postulate a synergistic action between neutrophilic and eosinophilic inflammation following an infectious/inflammatory stimulus. Notably, the upper lung areas would predominantly be spared from this excess of inflammation. It is known that the number of not only neutrophils, but also eosinophils, increases in the asthmatic airways during or after a viral infection. Epithelial cells in chronic asthma patients are damaged by granular products derived from eosinophils, such as MBP (major basic protein), and this increases the susceptibility to rhinovirus (RV) infection, which is one of the most frequent causes of asthma exacerbation [20]. RV enters cells via ICAM-1, leading to their increased expression on epithelial cells [25]. Nevertheless, ICAM-1 is also an adhesion and activation molecule of eosinophils, which are thus more activated by a rhinovirus infection [26]. In addition, there is often overexpression of the cytokine IL-8 in the airways in patients with severe asthma. The role of the cytokine IL-8 in determining neutrophil chemotaxis at sites of inflammation is well known [27]. It was shown in vitro that the neutrophils migrated for IL-8 were able to induce the transmembrane migration of eosinophils even without eosinophil chemotactic agents, but by means of LTB4 and PAF [27].

Finally, some recent investigations have shown that biological therapy with mepolizumab significantly reduced eosinophilic inflammation in severe asthmatic patients with bronchiectasis [28,29]. If the data observed in our small sample were to be confirmed by larger studies, we may speculate that, in patients with marked high blood eosinophilia, an appropriate biological therapy directed against the eosinophilic pathway could also slow down the eventual progression of bronchiectasis.

The most important limitations of our study are the small number of patients and its retrospective nature. Thus, further investigations with larger populations are mandatory to confirm our findings and to exclude a possible impact of parameters such as sex in the localization of bronchiectasis. Secondly, we only considered the location of the bronchiectasis as radiological parameter. Additional studies should also consider other radiological parameters potentially associated with elevated blood eosinophils.

Thirdly, we did not analyze eosinophils in sputum and/or in other respiratory matrices, and thus we were not able to correlate blood and sputum eosinophilia and their role in bronchiectasis, which is warranted in future investigations.

Fourthly, we did not quantify mucous plugs in the radiological study, which may have contributed to explain the basal distribution of bronchiectasis. Further investigations should mandatorily include assessment of mucous plugs.

Fifthly, we have focused on the localization of bronchiectasis and we have not collected quantitative descriptive parameters of bronchiectasis.

Finally, we did not include data about lymphocyte subpopulations which are necessary for future studies focused on the inflammatory profile of neutrophilic patients to highlight a possible contribution of neutrophilic exacerbations of bronchiectasis in their localization.

In conclusion, our data show an association between severe asthma patients with marked blood eosinophilia and coexisting non-CF and non-ASBA bronchiectasis with lower odds of upper lobe localization. Because of the small number of patients, our data cannot be generalized unless confirmed by larger patient cohorts.

## Figures and Tables

**Table 1:** Baseline features of the population.

Variables	Values
Demographics and lifestyle	
Age (y)	
median (interquartile range)	67.00 (56.75–71.00)
Female, % (n)	46.3 (19)
Smokers, % (n)	
currently	7.3 (3)
ex	36.6 (15)
never	56.1 (23)
BMI,	
m ± sd	25.24 ± 12.94
Asthma-related comorbidities	
Atopy % (n)	58.5 (24)
GER % (n)	39 (16)
Asa-sensitivity % (n)	9.8 (4)
AR % (n)	43.9 (18)
Atopic dermatitis % (n)	7.3 (3)
Nasal polyps % (n)	58.5 (24)
Chronic rhinosinusitis % (n)	53.7 (22)
Asthma Parameters	
Eos	
median (interquartile range)	625.00 (365.00–1140.00)
Eos > 500, %	70.7 (29)
Eos > 1000, %	26.8 (11)
IgE tot	
median (interquartile range)	252.00 (122.50–454.00)
FeNO50	
median (interquartile range)	38.00 (22.00–74.43)
Duration of asthma disease (y)	
median (interquartile range)	39.00 (20.00–54.00)
ACT	
median (interquartile range)	16.00 (11.25–18.00)
ACQ	
median (interquartile range)	3.20 (2.92–3.78)
% FEV1 l	
m ± sd	55.79 ± 17.48
% FVC	
m ± sd	79.64 ± 14.38
Bronchiectasis Parameters	
Basal localization bronchiectasis % (n)	78 (32)
Middle/lingular localization bronchiectasis % (n)	56.1 (23)
Upper localization bronchiectasis % (n)	41.5 (17)
Bilateral bronchiectasis % (n)	82.9 (33)
Widespread bronchiectasis % (n)	24.4 (10)
Number of lung segments with bronchiectasis	
median (interquartile range)	9 (5–15)
WBC	
median (interquartile range)	7400 (6890.00–10,640.00)

BMI: body mass index; ASA sensibility: acetylsalicylic acid sensibility; GERD: gastro-esophageal reflux disease; AR: allergic rhinitis; EOS: blood eosinophilia; FeNO: exhaled nitric oxide; FEV1: forced expiratory volume in the 1st second; FVC: forced vital capacity; ACT: asthma control test; ACQ: asthma control questionnaire; WBC: white blood cells.

**Table 2:** Comparison between two groups: Eos > 1000 vs. Eos ≤ 1000.

Variables	EOS ≤ 1000 n. Tot 30	EOS > 1000 n. Tot 11	*p* Value
Demographics			
Age (y) median (interquartile range)	65.5 (59.0–73.0)	54.0 (45.0–74.0)	0.175
Female, % (n)	36.7 (11)	72.7 (8)	0.075
Smokers, % (n)			0.965
currently	6.7 (2)	9.1 (1)	
ex	36.7 (11)	36.4 (4)	
never	56.7 (17)	64.5 (7)	
BMI, median (interquartile range)	25.00 (21.45–27.10)	24.30 (20.57–26.25)	0.452
Asthma-related Comorbidities			
Atopy % (n)	79.2 (24)	20.8 (2)	0.351
GER % (n)	36.4 (11)	40.0 (4)	0.564
Asa-sensitivity % (n)	10 (3)	9.1 (1)	0.712
Allergic rhinitis % (n)	46.7 (14)	36.4 (4)	0.411
Atopic dermatitis % (n)	10 (3)	0 (0)	0.381
Nasal polyps % (n)	45.5 (14)	64.3 (7)	0.250
Chronic rhinosinusitis % (n)	54.5 (16)	53.3 (6)	0.613
Asthma Parameters			
Eos median (interquartile range)	540.00 (300.00–630.00)	1350.00 (1277.5–1745.0)	
IgE tot median (interquartile range)	252.00 (120.00–746.00)	243.00 (129.25–378.50)	0.880
FeNO50 median (interquartile range)	38.00 (27.50–69.50)	76.00 (17.50–224.50)	0.948
Duration of asthma disease (y) median (interquartile range)	26.00 (12.00–39.50)	47.99 (18.27–54.50)	0.280
ACT median (interquartile range)	17 (11–20)	14.5 (12.25–17.50)	0.925
ACQ median (interquartile range)	3.0 (2.28–3.90)	3.15 (3.025–3.27)	0.351
%FEV1 l m ± sd	55.35 ± 17.04	57.10 ± 20.40	0.838
%FVC m ± sd	80.19 ± 14.08	78.10 ± 16.50	0.767
Bronchiectasis Parameters			
Basal localization bronchiectasis % (n)	80.0 (24)	72.7 (8)	0.456
Middle/lingular localization bronchiectasis % (n)	56.7 (17)	54.5 (6)	0.589
Upper localization bronchiectasis%	53.3 (16)	9.1 (1)	0.014
Bilateral bronchiectasis % (n)	73.2 (22)	26.8 (3)	0.069
Number of lung segments with bronchiectasis median (interquartile range)	9.0 (5.25–18.00)	8.00 (4.00–10.00)	0.147
WBC median (interquartile range)	7300.00 (6800.00–10,640)	7620.50 (7425.25–11,160.00)	0.192

BMI: body mass index; ASA sensibility: acetylsalicylic acid sensibility; GERD: gastro-esophageal reflux disease; AR: allergic rhinitis; EOS: blood eosinophilia; FeNO: exhaled nitric oxide; FEV1: forced expiratory volume in the 1st second; FVC: forced vital capacity; ACT: asthma control test; ACQ: asthma control questionnaire; WBC: white blood cells.

**Table 3:** Odds related to upper lung localization of bronchiectasis.

Variables	ODD (IC 95%)	*p* Value
Demographics and lifestyle		
Age (y)	1.011 (0.957–1.067)	0.704
Female, %	0.700 (0.200–2.454)	0.577
Smokers, % currently ex never	1.077 (0.402–2.882)	0.883
BMI, m ± ds median (interquartile range)	0.938 (0.807–1.091)	0.410
Asthma-related comorbidities		
Atopy	0.600 (0.167–2.162)	0.600
GER	0.764 (0.212–2.757)	0.681
Asa-sensitivity	0.438 (0.042–4.609)	0.491
AR	0.545 (0.152–1.955)	0.352
Atopic dermatitis	3.067 (0.255–36.878)	0.377
Nasal polyps	1.551 (0.432–5.570)	0.501
Chronic rhinosinusitis	0.420 (0.118–1.497)	0.181
Asthma Parameters		
Eos	0.999 (0.997–1.000)	0.119
Eos > 500, %	0.988 (0.252–3.870)	0.986
Eos > 1000, %	0.088 (0.010–0.772)	0.028
IgE tot	1.001 (1.000–1.001)	0.226
FeNO50	1.002 (0.991–1.012)	0.732
Duration of asthma disease (y)	0.978 (0.944–1.014)	0.225
ACT	1.104 (0.954–1.279)	0.185
ACQ	2.360 (0.831–6.702)	0.107
%FEV1 l	0.961 (0.913–1.012)	0.130
%FVC	0.983 (0.927–1.043)	0.983
Bronchiectasis Parameters		
WBC	1.001 (0.999–1.002)	0.611

BMI: body mass index; ASA sensibility: acetylsalicylic acid sensibility; GERD: gastro-esophageal reflux disease; AR: allergic rhinitis; EOS: blood eosinophilia; FeNO: exhaled nitric oxide; FEV1: forced expiratory volume in the 1st second; FVC: forced vital capacity; ACT: asthma control test; ACQ: asthma control questionnaire; WBC: white blood cells.

## Data Availability

Not applicable.
